# Supplementary material for: Processing of eukaryotic Okazaki fragments by redundant nucleases can be uncoupled from ongoing DNA replication in vivo
Source: Nucleic Acids Res. 2018 Dec 12;47(4):1814–22. doi: 10.1093/nar/gky1242 (PMC6393292; doi:10.1093/nar/gky1242)
Supplement: Supplementary Data [file gky1242_supplemental_files.pdf]

Kahli, Osmundson et al., Figure S1

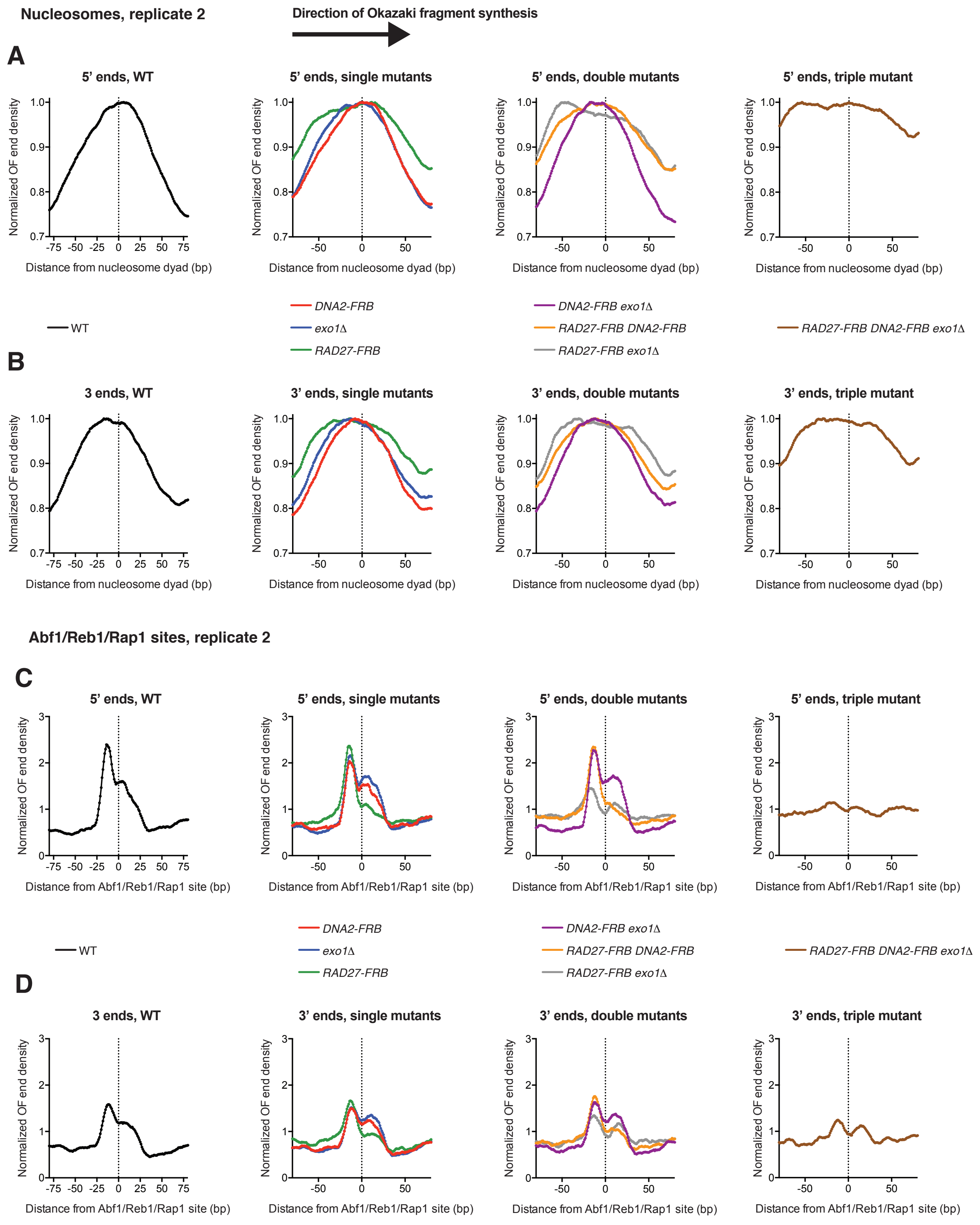

Figure S1. Sequencing data replicate comparisons

A. Distribution of Okazaki fragment 5' termini around nucleosome dyads, as in Fig. 2A, for a second biological replicate of each strain.
B. Distribution of Okazaki fragment 3' termini around nucleosome dyads, as in Fig. 2B, for a second biological replicate of each strain.
C. Distribution of Okazaki fragment 5' termini around Abf1/Reb1/Rap1 sites, as in Fig. 3A, for a second biological replicate of each strain.
D. Distribution of Okazaki fragment 3' termini around Abf1/Reb1/Rap1 sites, as in Fig. 3B, for a second biological replicate of each strain.

Kahli, Osmundson et al., Figure S2

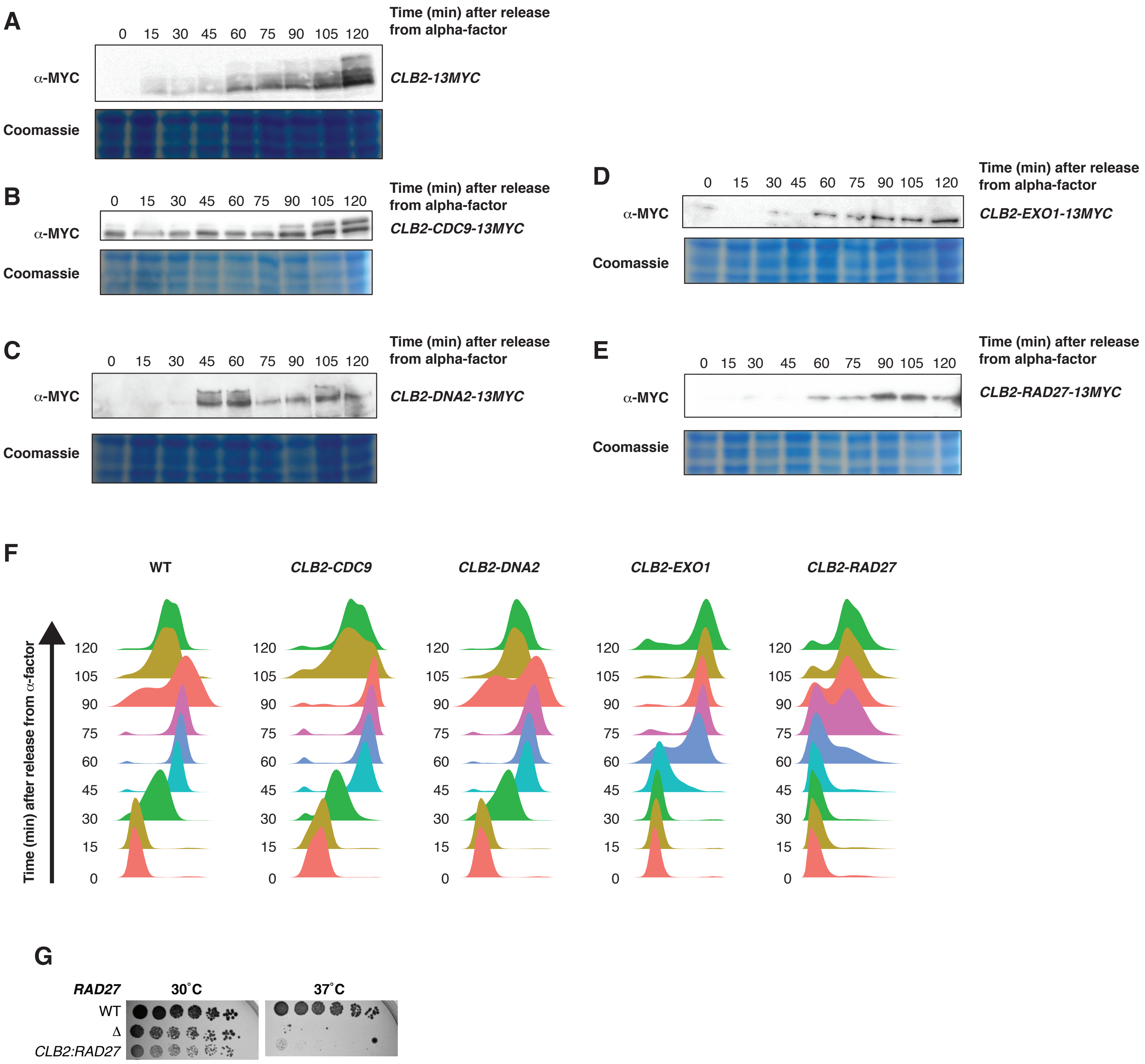

Figure S2. Expression and cell-cycle analysis for strains expressing Clb2-regulated Okazaki-fragment processing enzymes

A-E. Anti-Myc Western blots from strains released from alpha-factor arrest for the indicated time. CLB2 (A), CLB2-CDC9 (B), CLB2-DNA2 (C), CLB2-EXO1 (D) or CLB2-RAD27 (E) were C-terminally tagged with 13xMyc. Identically loaded Coomassie-stained gels are shown as loading controls. The lower band in (B) represents the mitochondrial isoform of Cdc9, which uses a distinct translational start site and does not cycle.
F. DNA content, assayed by flow cytometry, for samples analyzed in Fig. S2A-E
G. Spot tests on YPD at 30˚C or 37˚C, as indicated, comparing the growth of WT, *rad27*Δ and CLB2-RAD27 cells.
